# Interaction of the Human Papillomavirus E1 Helicase with UAF1-USP1 Promotes Unidirectional Theta Replication of Viral Genomes

**DOI:** 10.1128/mBio.00152-19

**Published:** 2019-03-19

**Authors:** Marit Orav, David Gagnon, Jacques Archambault

**Affiliations:** a Department of Microbiology and Immunology, McGill University, Montreal, Quebec, Canada; Yale School of Medicine; Icahn School of Medicine at Mount Sinai

**Keywords:** DNA replication, FA pathway, human papillomavirus

## Abstract

Human papillomaviruses (HPVs) are important pathogens with a significant medical burden. HPV genomes replicate in infected cells via bidirectional theta replication and a poorly understood unidirectional mechanism. In this report, we provide evidence that the previously described interaction between the viral E1 helicase and the cellular UAF1-USP1 deubiquitinating enzyme complex, a member of the Fanconi anemia DNA damage response pathway, is required for the completion of the bidirectional theta replication of the HPV11 genome and the subsequent initiation of the unidirectional replication. We show that unidirectional replication proceeds via theta structures and is supported by the cellular Bloom helicase, which interacts directly with E1 and whose engagement in HPV11 replication requires UAF1-USP1 activity. We propose that the unidirectional replication of the HPV11 genome initiates from replication fork restart events. These findings suggest a new role for the Fanconi anemia pathway in HPV replication.

## Abstract (precis)

Human papillomaviruses (HPVs) are important pathogens that replicate their double-stranded circular DNA genome in the nucleus of infected cells. HPV genomes replicate in infected cells via bidirectional theta replication and a poorly understood unidirectional mechanism, and the onset of viral replication requires the engagement of cellular DNA damage response pathways. In this study, we showed that the previously described interaction between the viral E1 helicase and the cellular UAF1-USP1 complex is necessary for the completion of bidirectional replication and the subsequent initiation of the unidirectional replication mechanism. Our results suggest HPVs may use the cellular Fanconi anemia DNA damage pathway to achieve the separation of daughter molecules generated by bidirectional theta replication. Additionally, our results indicate that the unidirectional replication of the HPV genome is initiated from restarted bidirectional theta replication forks.

## INTRODUCTION

HPVs infect keratinocytes of mucosal and cutaneous epithelia. While most HPV infections are asymptomatic or lead to the development of benign epithelial lesions, persistent infections with high-risk HPV types are associated with the development of several anogenital and head-and-neck cancers (1).

The circular 8-kbp double-stranded DNA (dsDNA) genome of HPVs is replicated in host cells as a multicopy episome. HPV replication depends on the expression of the viral E1 and E2 proteins (2–5) that control the sole origin of replication identified in the viral genome (6, 7). E1- and E2-dependent replication proceeds bidirectionally via theta structures (8–10); however, a unidirectional replication mechanism without a distinct origin sequence also participates in HPV replication (9, 10). During infection, three separate phases of replication occur—initial amplification of the viral genome upon entry of the HPV particle into the host cell, stable maintenance of HPV episomes in infected cells, and vegetative amplification of viral genomes prior to the production of progeny virions.

HPV E1 binds the cellular protein UAF1, an interaction that is conserved among mucosal HPV types (11). UAF1 is associated with three deubiquitinating enzymes, USP1, USP12, and USP46, and HPV E1 forms a ternary complex with UAF1 and any one of these three USPs (12). The E1-UAF1-USP complex is recruited to the viral origin and is necessary for efficient replication of the HPV genome (11–14). While the UAF1-USP12 and UAF1-USP46 complexes regulate histone deubiquitination (15) and the cell survival- and growth-promoting Akt pathway (16–20), the UAF1-USP1 complex is involved in the Fanconi anemia (FA) DNA repair pathway (21). HPV genome replication depends on the activation of both the ATM and ATR arms of the cellular DNA damage response (DDR) pathway (22–27). The FA pathway is mainly regulated by the ATR kinase (28, 29), and its dysfunction leads to extreme sensitivity to DNA interstrand crosslinks (ICLs). The many members of the FA pathway are classified into distinct groups based on their function. The FA targeting components, most notably FANCM, recruit the FA core complex to sites of DNA damage (28, 30). The core complex, in turn, ubiquitinates the FA ID complex (31–33) to promote its association with chromatin and its subsequent recruitment of FA effector proteins necessary for DNA repair (31, 34, 35). The FA ID complex is deubiquitinated by the UAF1-USP1 complex (21). Several links between the FA pathway and HPV replication have emerged in recent years. HPV E7 has been shown to activate the FA pathway and to upregulate the expression of several of its components (36–38). FANCD2, which is part of the FA ID complex, is recruited to HPV replication foci and binds to HPV episomes (38). The loss of FANCD2 impairs the replication and episomal maintenance of HPV genomes in undifferentiated keratinocytes (38) but contributes to enhanced replication during the productive part of the viral life cycle in differentiated keratinocytes (39). Episomal maintenance of HPV genomes also depends on the interaction between the HPV E2 protein and ChlR1 (40), a FANCJ-related cellular helicase that promotes the association of E2 with chromatin and is part of a recently discovered FA backup pathway involved in homologous recombination DNA repair (41).

The present study investigated the role of the E1-UAF1 interaction during the initial replicative amplification of the HPV11 genome. Our results show that efficient replication of HPV11 episomes in U2OS cells depends on the assembly of the E1-UAF1-USP1 ternary complex. We show that disruption of this complex by mutations or chemical inhibitors impedes the synthesis of viral replication intermediates (RIs) generated by the unidirectional mechanism more severely than those produced by bidirectional theta replication, with little to no effect on either bidirectional theta or unidirectional replication fork progression. Disruption of the complex had a more deleterious effect when viral DNA replication was stimulated by higher levels of E1 and E2. We also show that both bidirectional replication and unidirectional replication of HPV11 episomes proceed via theta structures and that the two replication mechanisms initiate from two different populations of HPV11 episomes. Lastly, we present evidence that E1 promotes unidirectional replication not only through its association with UAF1-USP1 but also by binding to the Bloom (BLM) helicase. We conclude that the E1-UAF1-USP1 complex supports the processing of late bidirectional theta RIs and propose that after the separation of bidirectional theta daughter genomes, the formerly converged replication forks undergo BLM-mediated restart, thus initiating the unidirectional theta replication of HPV genomes.

## RESULTS

### The E1-UAF1-USP1 complex is necessary for efficient replication of HPV11 episomes in U2OS cells.

**FIG 1:**
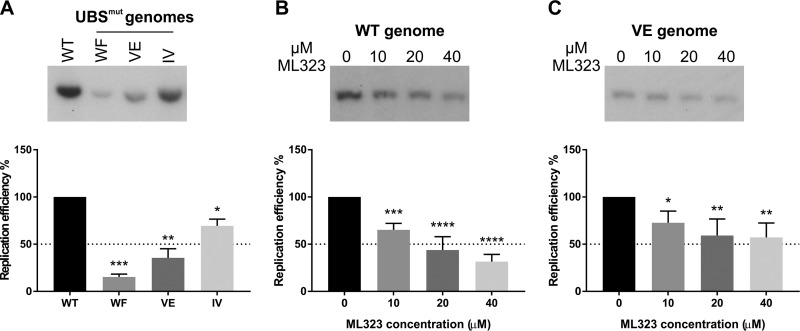
E1-UAF1-USP1 interaction is necessary for efficient replication of the HPV11 genome in U2OS cells. A Southern blot analysis of the transient replication of the HPV11 WT genome and of three UBS^mut^ genomes was performed (top), and a bar graph representing the replication efficiencies of the UBS^mut^ genomes relative to the replication efficiency of the WT genome is shown (bottom). Total cellular DNA was extracted from HPV11-transfected U2OS cells 72 h posttransfection and digested prior to analysis with the single-cutter restriction enzyme HindIII and the methylation-sensitive restriction enzyme DpnI. The bar graph data represent quantitated Southern blot signals from two independent experiments; error bars represent standard deviations. Statistical significance was determined using ordinary one-way ANOVA followed by Dunnett’s multiple-comparison test. (B and C) Southern blot analysis of the transient replication of HPV11 WT (B) or VE (C) genomes in the presence of increasing concentrations of ML323 (top); bar graph representing the effect of ML323 on the replication efficiency of the HPV11 WT (B) or VE (C) genome (bottom). ML323 (Millipore Sigma) or DMSO as a vehicle was added to the cell culture medium 24 h posttransfection. The DMSO concentration was constant for all ML323 dilutions and for the no-ML323 control. Total cellular DNA was extracted from HPV11-transfected U2OS cells 48 h posttransfection and linearized prior to analysis. Bar graphs represent quantitated Southern blot signals from four independent experiments; error bars represent standard deviations. Statistical significance was determined using ordinary one-way ANOVA followed by Dunnett’s multiple-comparison test.

Three double-alanine substitutions in the UAF1 binding site (UBS) of the HPV11 E1 protein have been shown to significantly impair its interaction with UAF1 (W17A/F18A, V20A/E21A, and I23A/V24A) (11). These three mutations were introduced into the full-length HPV11 genome, leading to the generation of the HPV11 UBS^mut^ WF, UBS^mut^ VE, and UBS^mut^ IV genomes. After transfection into U2OS cells, the wild-type (WT) HPV11 genome was replicated as a multicopy episome by the virally encoded E1 and E2 proteins, as shown previously (42) (Fig. 1A). All three UBS^mut^ genomes, in contrast, displayed reduced replication efficiency (Fig. 1A), achieving on average 15% (WF), 36% (VE), and 70% (IV) of the replication efficiency of the WT genome (Fig. 1A). The less severe replication defect of the UBS^mut^ IV genome likely reflects the residual UAF1-binding activity of the IV mutant E1 and is consistent with the effect of the analogous E1 mutation on the replication of HPV31 episomes in immortalized keratinocytes (13).

The interaction of E1 with UAF1 leads to the formation of a ternary complex containing E1, UAF1, and one of the three UAF1-associated deubiquitinating enzymes, USP1, USP12, or USP46 (12). ML323, a selective small-molecule inhibitor of the UAF1-USP1 complex (43), was used to test the requirement for USP1 activity in the replication of the HPV11 WT and VE mutant genomes. The VE genome was chosen for this and subsequent experiments as its replication efficiency is significantly lower than that of the WT genome, as a result of impaired E1-UAF1 binding, but is still sufficiently high to be measured accurately by Southern blotting. ML323 had a strong inhibitory effect on the replication of the WT genome, reaching 70% inhibition at 40 μM ML323 relative to the dimethyl sulfoxide (DMSO) vehicle control (Fig. 1B). At the same concentration, ML323 reduced the replication of the VE genome by approximately 40% (Fig. 1C). The lower sensitivity of the VE genome to inhibition by ML323 suggests that the loss of USP1 activity is intrinsic to the replication defect caused by abrogated UAF1 binding, consistent with all three proteins assembling into a ternary complex.

These results showed that the E1-UAF1 interaction is required for the replication of HPV11 episomes in U2OS cells, similarly to what has been observed previously for HPV31 in immortalized keratinocytes (11–14). Importantly, the results also revealed that the deubiquitinating activity of USP1 is essential for episomal replication of the HPV11 genome.

### The E1-UAF1-USP1 complex contributes to the generation of HPV11 RIs produced by unidirectional replication.

**FIG 2:**
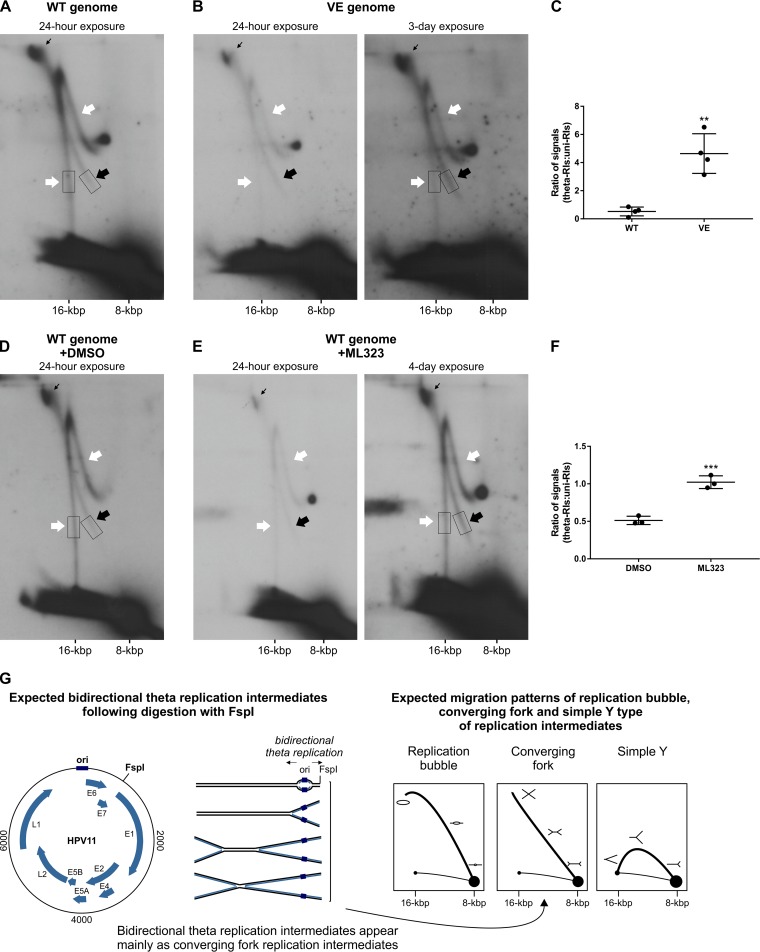
Disrupting the E1-UAF1-USP1 interaction specifically inhibits the unidirectional replication of the HPV11 genome. (A and B) 2D N/N AGE analysis of single-cutter restriction enzyme-digested RIs generated from the HPV11 WT genome (A) or VE genome (B). Low-molecular-weight DNA was extracted from HPV11-transfected U2OS cells 72 h posttransfection and digested with FspI. The results are representative of 4 experiments. Experiments were normalized to the number of transfected cells. White arrows mark uni-RIs, black arrows mark theta-RIs, and thin black arrows mark late theta-RIs. (D and E) 2D N/N AGE analysis of single-cutter restriction enzyme-digested RIs generated from the HPV11 WT genome in the presence of DMSO vehicle (D) or 20 µM ML323 (E). Low-molecular-weight DNA was extracted from HPV11-transfected U2OS cells 72 h posttransfection and digested with FspI. The results are representative of 3 experiments. Experiments were normalized to the number of transfected cells. White arrows mark uni-RIs, black arrows mark theta-RIs, and thin black arrows mark late theta-RIs. (C and F) Scatter graph depicting the ratio of signals representing theta-RIs and uni-RIs. Scatter graph data represent quantitated Southern blot signals; the areas used for quantitation are marked with boxes in panels A, B, D, and E. Each dot represents a separate experiment. The scatter graph represents quantitated signals from 4 (C) or 3 (F) separate experiments. Statistical significance was determined using unpaired Student's *t* tests; error bars represent the standard deviations. *P* = 0.0013, *t* = 5.701 (C); *P* = 0.0010, *t* = 8.679 (F). (G) Schematic overview of DNA molecules produced by FspI-digestion of RIs generated by bidirectional theta replication of HPV11 genomes, and their expected migration patterns during 2D N/N AGE (64).

To further characterize the impact of disrupting the E1-UAF1 interaction on HPV11 replication, two-dimensional (2D) neutral/neutral (N/N) agarose gel electrophoresis (AGE) was used to compare the RIs generated from the HPV11 WT and VE genomes (Fig. 2A and B). RIs were isolated from U2OS cells transfected with each genome 72 h posttransfection and linearized with the single-cutter restriction endonuclease FspI prior to analysis.

Exactly as observed for the HPV18 genome (10), two types of RIs were generated from the HPV11 WT genome that corresponded to those produced by bidirectional theta replication (theta-RIs; black arrows) and those generated by a unidirectional replication mechanism (uni-RIs; white arrows) (Fig. 2A). Digestion with FspI converted HPV11 theta-RIs into the converging fork type of RIs, which could be identified from their distinct migration pattern (Fig. 2G). Uni-RIs were identified based on their migration pattern, which is similar to that of RIs generated during the unidirectional replication of the HPV18 genome (10). Quantification of Southern blot signal strengths revealed that uni-RIs were twice as abundant as theta-RIs, as the average theta-RI/uni-RI ratio was 0.5 (Fig. 2C). Replication of the HPV11 UBS^mut^ VE genome gave rise to lower levels of RIs (Fig. 2B, 24-hour exposure), as anticipated from its decreased replication efficiency (Fig. 1A), but still produced both types of intermediates (Fig. 2B). Notably, however, the amount of uni-RIs was significantly decreased relative to theta-RIs such that for the VE genome, uni-RIs were no longer the predominant intermediates (theta-RI/uni-RI ratio of 4.6; Fig. 2C). Inspection of the migration patterns of both theta- and uni-RIs generated from the HPV11 VE genome revealed no evidence of replication fork stalling (Fig. 2B), which would have presented as discrete spots along the arcs traced by each RI type (44). The only RIs that accumulated during replication of the HPV11 WT and VE genomes were late bidirectional theta RIs corresponding to almost fully replicated but not yet separated genomes (thin black arrows, Fig. 2A and B).

To determine the effect of inhibiting the UAF1-USP1 interaction on HPV11 RIs, 2D N/N AGE analysis of RIs generated from the WT genome in the presence of 20 µM ML323 was performed (Fig. 2E). As anticipated, in the presence of DMSO, uni-RIs (Fig. 2D, white arrows) remained more prevalent than theta-RIs (Fig. 2D, black arrows), indicating little to no effect of the vehicle on the replication of WT episomes (theta-RI/uni-RI ratio of 0.51; Fig. 2F). In the presence of 20 μM ML323, however, the prevalence of uni-RIs was significantly decreased (Fig. 2E), resulting in nearly equal abundances of theta- and uni-RIs (theta-RI/uni-RI ratio of 1.07; Fig. 2F). The effect of inhibiting the UAF1-USP1 interaction was very similar to that of inhibiting the E1-UAF1 interaction, confirming that the loss of USP1 activity is essential to the replication defect caused by abrogated UAF1 binding.

In summary, the results presented above indicate that inhibiting the E1-UAF1-USP1 interaction is more detrimental to the generation of uni-RIs than theta-RIs, revealing that USP1 activity is necessary for unidirectional replication. As disruption of the E1-UAF1-USP1 interaction has no major effect on fork progression, we surmise that it is necessary for the initiation of unidirectional replication but dispensable during the elongation phase of DNA synthesis.

### HPV11 genomes give rise to similar RIs in U2OS and HaCaT cells.

**FIG 3:**
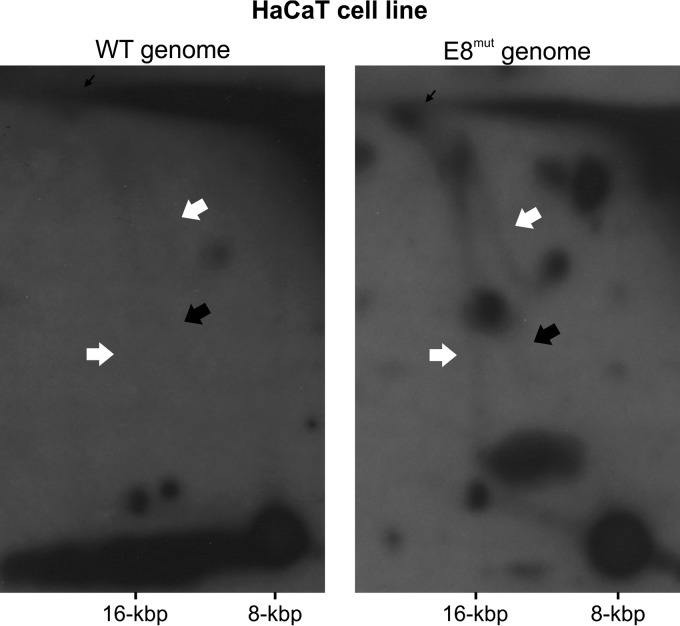
HPV11 RIs produced in the HaCaT cell line are identical to those generated in U2OS cells. 2D N/N AGE was performed with single-cutter restriction enzyme-digested RIs generated from the HPV11 WT and E8^mut^ genomes in HaCaT cells. Low-molecular-weight DNA was extracted from HPV11-transfected HaCaT cells 72 h posttransfection and digested with FspI. Experiments were normalized to the number of transfected cells. White arrows mark uni-RIs, black arrows mark theta-RIs, and thin black arrows mark late theta-RIs.

To confirm that RIs generated from HPV11 genomes in U2OS cells represent *bona fide* HPV11 RIs, we repeated the 2D N/N AGE of RIs generated from HPV11 WT and E8^mut^ genomes in HaCaT cells (Fig. 3). The HaCaT cell line was established from spontaneously immortalized keratinocytes and maintains full epidermal differentiation capability (45), thus representing a more natural environment for HPV replication. The HPV11 E8^mut^ genome lacks the expression of the E8^E2 protein, a potent repressor of both HPV transcription and replication (reviewed in reference 46), thus displaying enhanced replication levels (47). RIs were extracted from transfected HaCaT cells 72 h posttransfection and linearized with FspI prior to analysis (Fig. 3).

HaCaT cells supported HPV11 replication at a much lower level of efficiency than U2OS cells. As a result, RIs generated from the HPV11 WT genome were barely detectable by 2D N/N AGE (Fig. 3), while RIs generated from the poorly replicating HPV11 VE genome were too scarce to be detected (data not shown). In contrast, analysis of the HPV11 E8^mut^ genome revealed the presence of both theta- and uni-RIs similar to those observed in U2OS cells (Fig. 3).

These results indicated that theta- and uni-RIs represent *bona fide* HPV11 RIs and validated the use of U2OS cells for the characterization of HPV11 replication products and intermediates.

### Increased expression of HPV11 E1 and E2 exacerbates the replication defect of UBS mutant genomes.

**FIG 4:**
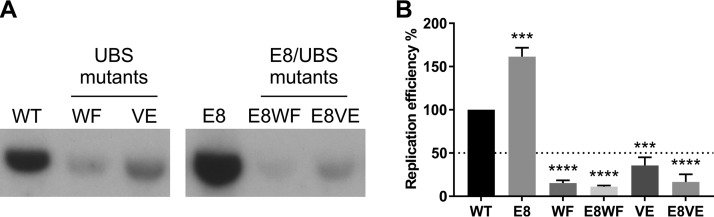
Eliminating the expression of the E8^E2 repressor protein further decreases the replication efficiency of HPV11 genomes unable to bind UAF1. (A) Southern blot analysis of total DNA extracted from U2OS cells transfected with HPV11 WT, UBS^mut^, E8^mut^, and E8^mut^UBS^mut^ genomes. Total cellular DNA was extracted from HPV11-transfected U2OS cells 72 h posttransfection and digested prior to analysis with the single-cutter restriction enzyme HindIII and the methylation-sensitive restriction enzyme DpnI. The results are representative of 2 experiments. (B) Bar graph representing the replication efficiencies of different HPV11 genomes relative to the replication efficiency of the WT genome. Data represent quantitated Southern blot signals from 2 separate experiments; statistical significance was determined using ordinary one-way ANOVA followed by Dunnett’s multiple-comparison test. Error bars represent the standard deviations.

To test whether the negative effect of disrupting the E1-UAF1 complex can be rescued by elevated E1 and E2 levels, a mutation eliminating the expression of the E8^E2 transcriptional repressor was introduced into the HPV11 UBS^mut^ WF and VE genomes (Fig. 4). UBS^mut^ IV was excluded from this analysis due to its suspected residual UAF1-binding activity. In U2OS cells, the replication efficiency of the E8^E2 (E8) mutant genome was on average 1.6-fold higher than that of the WT genome (Fig. 4B, compare WT and E8). When the E8 mutation was introduced into the WF and VE mutant genomes, however, it further decreased the replication efficiency of those mutants (Fig. 4; compare WF and E8WF and compare VE and E8VE). For example, the replication efficiency of the E8VE genome was on average 17% of that of the WT genome, a 2-fold decrease from the replication efficiency of the VE genome (36% of the WT genome) (Fig. 4B). Thus, the E8 mutation exacerbates rather than alleviates the replication defect of HPV11 UBS^mut^ genomes.

Higher expression of E1 and E2 should increase bidirectional theta replication by stimulating its initiation from the sole E1- and E2-dependent replication origin present in the HPV11 genome but would be expected to have little to no effect on the unidirectional replication mechanism, which initiates independently of a distinct replication origin sequence (9, 10). As such, these results suggest that increased level of bidirectional theta replication initiation aggravates the replication defect of HPV11 UBS mutant genomes.

### Bidirectional theta replication and unidirectional replication are initiated from HPV11 genomes in different topological forms.

**FIG 5:**
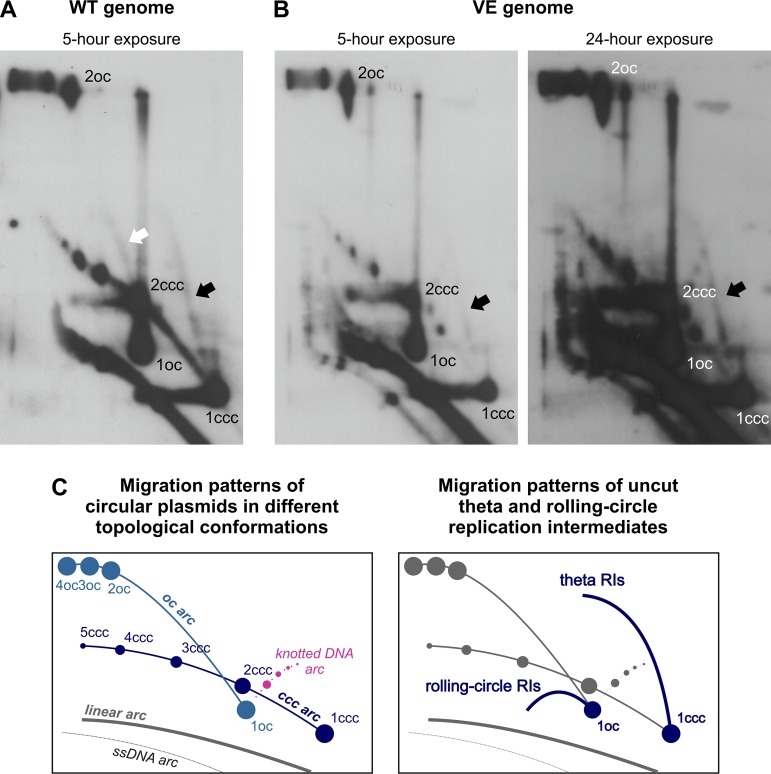
RIs generated by bidirectional theta replication and unidirectional replication arise from HPV11 genomes in different topological forms. (A and B) 2D N/N AGE analysis of uncut RIs generated from the HPV11 WT (A) or VE (B) genomes. Low-molecular-weight DNA was extracted from HPV11-transfected U2OS cells 72 h posttransfection and digested prior to analysis with the methylation-sensitive restriction enzyme DpnI to remove input HPV11 genomes. The results are representative of 2 experiments. Experiments were normalized to the number of transfected cells. White arrow marks uni-RIs, and black arrows mark theta-RIs. (C) Schemes depicting the expected migration patterns of circular plasmids in different topological conformations (left) and uncut theta and rolling-circle RIs (right).

Previous findings regarding HPV18 (10) and those presented thus far for HPV11 suggest that bidirectional replication and unidirectional replication are distinct but interdependent modes of viral DNA synthesis. To investigate this possibility further, we sought to identify the DNA substrates from which each mechanism is initiated. To do so, 2D N/N AGE analysis of undigested DNA extracted from U2OS cells transfected with the HPV11 WT and VE genomes was performed (Fig. 5), as uncut DNA analysis is especially helpful for characterizing circular molecules and RIs arising from circular genomes.

Predictably, the HPV11 WT genome gave rise to two groups of RIs (Fig. 5A, marked with arrows). One group emanated from the monomeric 8-kbp HPV11 genomes in covalently closed circular topological form (1ccc) (Fig. 5A, black arrow). The other seemed to originate from either monomeric 8-kbp genomes in open circular topological form (1oc) or covalently closed dimeric 16-kbp (2ccc) HPV11 genomes (Fig. 5A, white arrow). Given the similar migration patterns of the two RI groups, which indicated that they were of similar shape and size (48), it is likely that the second group of RIs emanated from open circular monomeric 8-kbp genomes. Both groups of RIs traced a migration pattern that is indicative of theta-like replication (48), and the presence of the rolling-circle type of replication could be ruled out as no RIs consistent with this mechanism were observed (48, 49) (Fig. 5C). The HPV11 VE genome gave rise to only one detectable group of RIs emanating from monomeric 8-kbp covalently closed circular HPV genomes (Fig. 5B, black arrow). Since replication of the HPV11 VE genome generated more bidirectional theta-RIs than uni-RIs (Fig. 2B), this finding identifies the 8-kbp covalently closed circular HPV11 genomes as the population replicated by bidirectional theta replication (Fig. 5A and B, black arrows). The RIs generated from the open circular monomeric 8-kbp genomes disappeared when the E1-UAF1 interaction was abrogated (compare Fig. 5A, white arrow, and Fig. 5B), suggesting that they represent uni-RIs, which are known to be inhibited by the interruption of UAF1 binding.

These results indicate that theta- and uni-RIs arise from two different populations of HPV11 genomes. Theta-RIs are generated from covalently closed monomeric 8-kbp genomes, while the unidirectional replication mechanism most likely initiates from open circular monomeric 8-kbp HPV11 genomes. HPV dsDNA genomes are in open circular topological form when they contain single-stranded DNA (ssDNA) nicks or gaps, so this topological form would be adopted by monomeric HPV genomes arising from the separation of almost fully replicated late bidirectional theta RIs. This, together with previous data, raises the possibility that the E1-UAF1-USP1 interaction is involved in achieving the separation of HPV11 daughter molecules generated via bidirectional theta replication and that the unidirectional replication mechanism is subsequently initiated from those newly separated molecules.

### HPV11 E1 protein interacts with BLM helicase *in vitro*.

**FIG 6:**
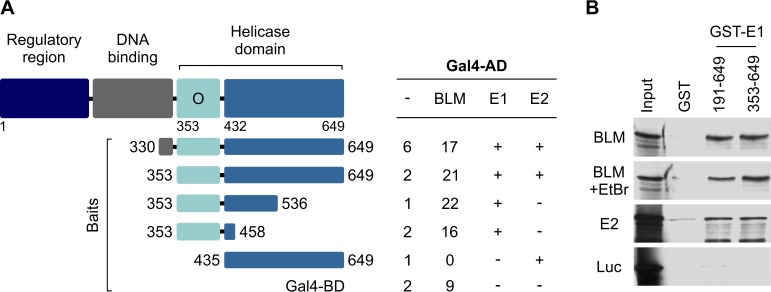
HPV11 E1 protein interacts with BLM helicase. (A) Yeast two-hybrid screen for E1 interaction partners. A diagram of HPV11 E1 indicating the positions of the N-terminal regulatory region, DNA-binding domain (BD), and C-terminal helicase domain is shown. The minimal region required for E1 oligomerization in yeast cells is marked with “O.” The amino acid sequence boundaries of each bait are indicated on the left; levels of measured β-galactosidase activity are indicated on the right. The ability of different baits to interact with E1 and E2 is indicated as a control. AD, activation domain. (B) GST pull-down assay demonstrating *in vitro* interaction between HPV11 E1 and BLM. Two different GST-tagged E1 fragments were used in the experiment: one fragment spanning E1 amino acid sequences from position 191 to position 649 and the other fragment spanning sequences from position 353 to position 649. An EtBr control was included to check for DNA contamination. E2 was used as a positive control and luciferase enzyme as a negative control for *in vitro* interaction.

Yeast two-hybrid screening of a human lymphocyte cDNA library identified a novel interaction between HPV11 E1 and the cellular BLM helicase (Fig. 6A). The BLM helicase plays a critical role in cellular DDR and has been implicated in the FA pathway-mediated restart of stalled replication forks (50, 51). The fragment of BLM helicase shown to interact with E1 in the two-hybrid system spans amino acid positions 1 to 514 (data not shown) and is known to facilitate interactions between BLM and its binding partners, such as Rad51 (52) and replication protein A (RPA) (53). Using a series of truncated baits, the region of E1 necessary for BLM interaction was mapped to a region encompassing the minimal oligomerization domain between amino acid positions 353 and 431 (54) (Fig. 6A). The ability of the truncated E1 fragments used as bait to interact with HPV11 E1 (i.e., to support E1 oligomerization) and with the transactivation domain of E2 has been previously reported (54, 55) and is indicated as a control (Fig. 6A). Two fragments of E1 purified as glutathione *S*-transferase (GST) fusion proteins were then tested in a pull-down assay for interaction with *in vitro*-translated BLM, using *in vitro*-translated HPV11 E2 and firefly luciferase as a positive control and a negative control, respectively (Fig. 6B). Ethidium bromide (EtBr) was included in the binding reactions to rule out the possibility that the E1-BLM interaction is mediated by contaminating DNA (Fig. 6B). Unlike GST alone, both GST-E1 proteins were able to pull down BLM and E2 but not luciferase (Fig. 6B). Collectively, these results indicate that HPV11 E1 can specifically interact with the BLM helicase *in vitro*.

### The generation of uni-RIs is supported by the BLM helicase.

**FIG 7:**
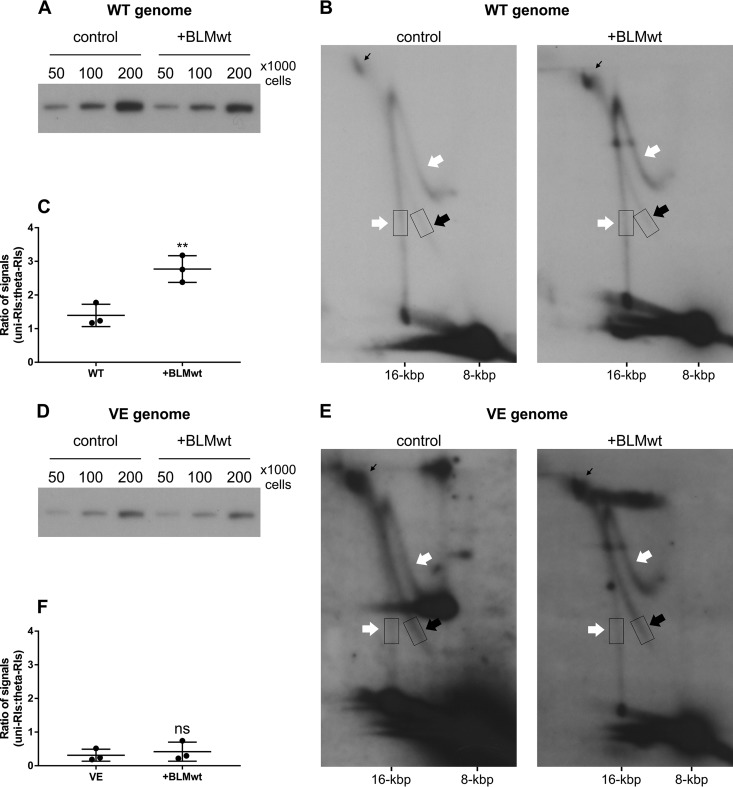
BLM helicase stimulates the generation of uni-RIs. (A and D) 1D AGE analysis of the overall replication efficiency of the HPV11 WT genome (A) or VE genome (D) in the absence (control) or presence (+BLMwt) of wild-type BLM overexpression. Low-molecular-weight DNA was extracted from HPV11-transfected U2OS cells 72 h posttransfection and normalized to cell count. The number of cells per sample is indicated. Samples were digested with the single-cutter endonuclease HindIII and the methylation-sensitive restriction enzyme DpnI. The results are representative of 2 experiments. (B and E) 2D N/N AGE analysis of RIs generated from the HPV11 WT genome (B) or VE genome (E) in the absence (control) or presence (+BLMwt) of wild-type BLM overexpression. Low-molecular-weight DNA was extracted from HPV11-transfected U2OS cells 72 h posttransfection and digested prior to analysis with the single-cutter endonuclease FspI and the methylation-sensitive restriction enzyme DpnI. The results are representative of 3 experiments. Experiments were normalized to the number of transfected cells. White arrows mark uni-RIs, black arrows mark theta-RIs, and thin black arrows mark late theta-RIs. (C and F) Scatter graph depicting the ratio of signals representing uni-RIs and theta-RIs. Scatter graph data represent quantitated Southern blot signals from 3 separate experiments for each data set; areas used for quantitation are marked with boxes in panels B and E. Each dot represents a separate experiment. Statistical significance was determined using an unpaired Student's *t* test, and error bars represent the standard deviations. *P* = 0.0100, *t* = 4.605 (C); *P* = 0.6102, *t* = 0.5522 (F).

Given that BLM interacts with E1, we examined the effect of overexpressing BLM helicase on RIs generated from the HPV11 WT and VE genomes (Fig. 7).

BLM overexpression did not affect the overall replication efficiency of the HPV11 WT (Fig. 7A) or VE mutant (Fig. 7D) genome. However, 2D N/N AGE analysis revealed that overexpression of BLM increased the prevalence of uni-RIs generated from HPV11 WT episomes (Fig. 7B, white arrows). Quantitation of Southern blot signal strengths indicated that the average uni-RI/theta-RI ratio was increased almost 2-fold, from 1.40 to 2.77, in the presence of BLM overexpression (Fig. 7C). Similar experiments performed with the HPV11 VE genome showed that the relative abundances of uni-RIs and theta-RIs were unchanged by overexpression of BLM (Fig. 7E). Quantitation of Southern blot signal strengths indicated that the ratio of signals representing uni-RIs and theta-RIs rose from 0.31 to 0.42 in the presence of BLM overexpression; however, this change was not statistically significant (Fig. 7F). Overexpression of two dominant-negative BLM helicases, a helicase-dead mutant and a sumoylation mutant, led to an overall decrease in the levels of both theta-RIs and uni-RIs (data not shown), consistent with a general inhibition of HPV DNA replication.

Collectively, these results indicate that the E1-BLM interaction promotes the production of uni-RIs, supporting the notion that uni-RIs are generated from the BLM-mediated restart of bidirectional theta replication forks after the separation of almost fully replicated bidirectional theta replication intermediates. Importantly, BLM overexpression is unable to support unidirectional replication in the absence of E1-UAF1 binding, indicating that USP1 activity is required to engage BLM in HPV11 replication.

## DISCUSSION

**FIG 8:**
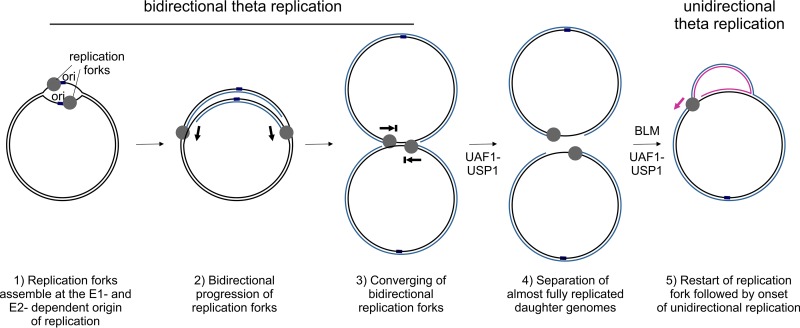
The proposed involvement of the UAF1-USP1 complex and BLM helicase in HPV11 replication.

This study investigated the mechanism through which the interaction between HPV11 E1 and the cellular UAF1-USP1 deubiquitinating complex supports viral replication. We confirmed that HPV11 genome replication gives rise to two distinct groups of intermediates similar to those previously described for HPV16 (9) and HPV18 (10). These two groups correspond to RIs generated by bidirectional theta replication and a unidirectional replication mechanism without a specific origin sequence (9, 10) (Fig. 2). Similarly to HPV18 (10), RIs indicative of these two replication mechanisms were generated from HPV11 genomes in both the U2OS and HaCaT cell lines (Fig. 2 and 3). Also, the previously described transcription map of the HPV11 genome in U2OS cells is similar to the transcription map of the HPV11 genome in HPV-associated lesions (47), further validating the use of the U2OS cell line to study the replication of HPV episomes. The importance of the E1-UAF1-USP1 complex for efficient HPV replication has been previously reported (11–14); however, we demonstrated that the lower replication efficiency of mutant HPV11 genomes encoding a E1 protein defective for UAF1-binding is associated with decreased production of RIs generated by the unidirectional mechanism, while bidirectional theta replication was affected to a lesser degree (Fig. 2). Importantly, we also showed that the replication defect caused by the abrogation of the E1-UAF1 interaction was largely due to the loss of the deubiquitinating activity of USP1 (Fig. 1 and 2). We present evidence for two interlinked processes that require USP1 activity and are necessary for the onset of unidirectional HPV11 replication. A schematic depiction of the involvement of the E1-UAF1-USP1 interaction in HPV replication is presented in Fig. 8.

First, our results indicated that the E1-UAF1-USP1 interaction is necessary to process late bidirectional theta RIs that accumulate during the replication of the viral genome. This would explain why elevated E1 and E2 expression exacerbated the replication defect of the UBS^mut^ genomes (Fig. 4). Overexpression of E1 and E2 is expected to increase the initiation of bidirectional replication from the sole E1- and E2-dependent origin of replication (6, 7); thus, enhanced bidirectional replication would increase the burden of molecules in need of resolution and exacerbate the replication defect of UBS^mut^ HPV11 genomes. Additionally, a role for the E1-UAF1-USP1 complex in the processing of late bidirectional theta RIs would explain why defective E1-UAF1-USP1 complex formation has no effect on the progression of theta and unidirectional replication forks (Fig. 2). How HPVs achieve the separation of their newly replicated circular genomes is unknown, and no cellular or viral factors involved in the process have been identified. Late theta RIs are structurally similar to ICLs, which are processed by the FA pathway in cells (56). The UAF1-USP1 complex plays a critical role in the FA DNA repair pathway, and it is possible that HPV11 RIs containing two converged theta replication forks are mistaken for ICLs and that the FA pathway is recruited to resolve the structures.

Second, we provided evidence that the unidirectional replication of HPV11 genomes is initiated from newly separated daughter molecules generated by bidirectional theta replication via BLM-mediated replication fork restart events. The unidirectional replication mechanism was initiated from monomeric HPV11 genomes in open circular topological form (Fig. 5). Such molecules are expected to arise from the separation of almost fully replicated bidirectional theta daughter molecules. The converging of replication forks would prevent the replication of a small portion of the HPV genome, giving rise to an ssDNA gap in the daughter molecules and leading to the adoption of the open circular topological form. Conversely, bidirectional theta replication was initiated from monomeric HPV11 genomes in covalently closed circular topological form, consistent with either HPV11 genomes transfected into U2OS cells or end products of fully completed replication (Fig. 5). Additionally, we showed that HPV11 E1 interacted with BLM helicase *in vitro* (Fig. 6) and that BLM overexpression promoted the generation of RIs produced by the unidirectional mechanism (Fig. 7). Cellular BLM helicase has been shown to participate in the restart of stalled or collapsed replication forks (57, 58).

Several characteristics of the unidirectional replication mechanism, such as the lack of a distinct origin sequence (9, 10) and its onset being subsequent to the onset of bidirectional theta replication (10), correspond to initiation from restarted theta replication forks. Additionally, the unidirectional replication mechanism gives rise to replication forks with opposing polarities that traverse the viral genome in both directions (10), also supporting the notion of initiation from restarted theta replication forks. We showed that, similarly to bidirectional replication, the unidirectional replication of HPV11 genomes proceeded via theta structures (Fig. 5). We were unable to detect RIs characteristic of the rolling-circle type of replication such as sigma-RIs or circular ssDNA molecules (48, 49), indicating that this particular mechanism is not involved in the initial amplification of HPV11 genomes (Fig. 5). Previous analysis indicated that uni-RIs have a very complex shape (10) that is suggestive of the presence of unknown structural elements in addition to a single unidirectional replication fork. The nature of those additional structural elements is unknown, and any corresponding suggestions, such as those involving the presence of X-shaped DNA junctions, are merely speculative.

The results presented in this paper also revealed clues as to how the interaction between E1 and the UAF1-USP1 complex might lead to the BLM-mediated initiation of unidirectional replication. Our results indicated that the E1-BLM interaction depends on the presence of the minimal E1 amino acid sequence required for E1 oligomerization (54) (Fig. 6A), suggesting that BLM is recruited to hexameric E1 replication forks. Additionally, the ability to engage BLM in HPV11 replication depended on USP1 activity, as the overexpression of BLM was unable to support the unidirectional replication of HPV11 genomes defective for UAF1-binding (Fig. 7E and F). FANCD2 has been shown to cooperate with the BLMcx complex to promote the restart of stalled replication forks while suppressing the initiation of replication from nonactive replication origins (50, 51). These functions of FANCD2 are independent of the FA core complex and involve deubiquitinated FANCD2 (51). Links between FANCD2 and HPV replication, including the recruitment of FANCD2 to HPV replication centers, have been described previously (38, 39). When the cellular FA pathway is activated, the ubiquitination of FA ID complex members FANCI and FANCD2 by the FA core complex leads to the recruitment of the FA effector proteins and DNA repair. By recruiting the UAF1-USP1 complex to HPV replication forks, HPV11 may promote the deubiquitination of FANCD2 bound to viral genomes, thus promoting the engagement of BLM over the FA effector proteins.

The data presented in the present manuscript help to elucidate two important aspects of HPV replication. First, the separation of daughter molecules generated via bidirectional theta replication. Currently, almost nothing is known about how the virus completes the replication of its circular genome. Second, we propose that the unidirectional replication mechanism involved in HPV replication is initiated from those newly separated daughter genomes via BLM-mediated restart of bidirectional theta replication forks, thus identifying the mechanism underlying that mode of replication. Collectively, our findings provide evidence that HPV11 recruits the cellular FA pathway to process the end products of bidirectional theta replication, which in turn leads to initiation of the unidirectional replication of the HPV11 genome.

## MATERIALS AND METHODS

### Cell lines and transfections.

U2OS and HaCaT cells were grown in Iscove’s modified Dulbecco’s medium (IMDM) supplemented with 10% fetal bovine serum, 100 U/ml penicillin, and 0.1 mg/ml streptomycin at 37°C at 5% CO_2_. U2OS and HaCaT cells were transfected through electroporation using a Bio-Rad Gene Pulser Xcell supplied with a capacitance extender (Bio-Rad Laboratories) at 200 V and the capacitance set to 975 μF. U2OS cells were transfected with 2 μg of the appropriate HPV11 genome, except for the experiments performed with the inhibitor ML323 (Fig. 1B and C), when U2OS cells were transfected with either 0.5 μg of the WT HPV11 genome or 3 μg of the VE HPV11 genome. HaCaT cells were transfected with 5 µg of the appropriate HPV11 genome. BLM helicase overexpression was achieved by cotransfecting U2OS cells with 1 µg of BLM expression plasmid.

### Plasmids.

All HPV11 full-length genomes were produced as minicircle plasmids in covalently closed circular topological form (59). In brief, the minicircle production vector pMC.BESPX was inserted into the BamHI restriction site of the HPV11 genome at position 7072. Minicircle HPV11 genomes were purified from Escherichia coli minicircle producer ZYCY10P3S2T cells following the instructions provided previously (60). Mutations abrogating the interaction between HPV11 E1 and UAF1 have been previously described (11). In brief, mutations leading to double amino acid substitutions at E1 amino acid positions 17 to 18 (WF to AA), 20 to 21 (VE to AA), and 23 to 24 (IV to AA) were introduced into the full-length HPV11 genome to generate the HPV11 UBS^mut^ WF, VE, and IV genomes, respectively. The HPV11 E8 mutant genome has been described previously (47). In brief, a T-to-C substitution was introduced at nucleotide position 1242 of the HPV11 genome, changing the ATG start codon of the E8 reading frame to ACG. Expression vectors for wild-type Bloom helicase and the Bloom sumoylation mutant (described in references 61 and 62) were a gift from Nathan Ellis (Addgene plasmids 80070 and 80071). The helicase-dead dominant-negative ATPase mutant Bloom helicase contains a K695A substitution. The HPV11 UBS^mut^ genomes and the helicase-dead mutant Bloom helicase were created by Civic Bioscience Ltée.

### 1D AGE.

Total cellular DNA was extracted from HPV11-transfected U2OS cells with Qiagen’s DNeasy blood & tissue kit at 48 (Fig. 1B and C) or 72 (Fig. 1A and 3A) hours posttransfection. Alternatively, low-molecular-weight DNA was extracted from HPV11-transfected U2OS cells using the Hirt method (60) 72 h posttransfection (Fig. 7A and D). DNA samples were digested with single-cutter restriction enzyme HindIII (New England Biolabs) and methylation-sensitive restriction enzyme DpnI (New England Biolabs) at 37°C for 2 h prior to analysis. 1D AGE was run using a 1% agarose gel submerged in 1× Tris-acetate-EDTA (TAE) buffer at 1.1 V/cm for 22 h at room temperature.

### 2D N/N AGE.

Low-molecular-weight DNA was extracted from HPV11-transfected U2OS or HaCaT cells using the Hirt method (60) 72 h posttransfection. Extracted DNA was digested with the single-cutter restriction enzyme FspI (New England Biolabs) overnight at 37°C for the analysis of digested RIs (Fig. 2, 3, and 7). The 2D N/N AGE technique has been extensively described previously (60). The first dimension was run using a 0.4% agarose gel submerged in 0.5× Tris-borate-EDTA (TBE) buffer either at 0.8 V/cm (digested DNA) or at 0.65 V/cm (uncut DNA) at room temperature for 22 h. The second dimension was run using either a 1% (digested DNA) or 0.6% (uncut DNA) agarose gel submerged in 0.5× TBE buffer at 6 V/cm at 4°C for 5 h.

### Southern blotting.

DNA was transferred from the agarose gel to an Amersham Hybond-XL filter (GE Healthcare) using either capillary transfer (described previously [60]) or a Bio-Rad Model 785 vacuum blotter (Bio-Rad Laboratories). The subsequent hybridization method was described previously (60). Radioactively labeled HPV11 genomic probes were generated using a DecaLabel DNA labeling kit (Thermo Fisher Scientific) and [α-^32^P]dCTP (PerkinElmer).

### Southern blot signal quantitation and statistics.

Southern blot signals were quantitated with Image Studio Lite Ver 5.2. For 2D N/N AGE analyses, the approximate areas used to quantitate Southern blot signals representing RIs generated by bidirectional theta and unidirectional replication are marked in each figure with boxes. All data were analyzed with GraphPad Prism 7. The statistical analysis of signal ratios calculated for 2D N/N AGE analyses used the Student's *t* test, while the remaining statistical analyses used ordinary one-way analysis of variance (ANOVA) followed by Dunnett’s multiple-comparison test. All *P* values (*, *P* ≤ 0.05; **, *P* ≤ 0.01; ***, *P* ≤ 0.001; ****, *P* ≤ 0.0001) are two-tailed.

### Yeast two-hybrid system and GST pull-down assay.

Construction of the E1 bait proteins was described previously (55). Briefly, PCR-amplified fragments of the E1 open reading frame were inserted into the pAS1 plasmid containing the DNA-binding domain of GAL4. The baits were screened against a human lymphocyte cDNA library cloned into the pACT2 vector backbone containing the activation domain of GAL4. The yeast two-hybrid screening procedure has been described previously (55). For the GST pull-down assay, DNA sequences coding for the E1 amino acid sequences from position 353 to 649 and position 191 to 649 were GST tagged, inserted into the pFASTBAC1 expression vector (Thermo Fisher Scientific), expressed, and purified as previously described (63). The BLM, E2, and luciferase proteins used in GST pull-down assays were generated by *in vitro* transcription-translation using a TnT quick-coupled transcription/translation system (Promega). The T7 promoter-driven plasmids used for *in vitro* transcription-translation were generated as previously described (55), while the plasmid for luciferase was included in the kit. The pull-down assay was performed as previously described (55).
